# Performance and physical activity. How playful activities implemented into training in Danish esports clubs influence players' performance experience

**DOI:** 10.3389/fspor.2024.1441607

**Published:** 2024-12-03

**Authors:** Lars Domino Østergaard, Christian Lund Nørgaard Straszek, Lasse Nørgaard Frandsen

**Affiliations:** ^1^ Department of Health Science and Technology, Aalborg University, Aalborg, Denmark; ^2^ Department of Physiotherapy, University College of Northern Denmark, Aalborg, Denmark

**Keywords:** exercise, gaming, gamer skills, coaching, Counter-Strike: Global Offensive

## Abstract

**Introduction:**

Over the past few years, attention has focused on how physical activity can enhance esports players' performance. For example, complementing esports training with physical activities has been explored. However, most of these activities are based on traditional strength or endurance-related exercises, which do not align with the interests of children attending organized esports clubs.

**Methods:**

In this study, we investigate playful physical activities. Our research is based on qualitative pilot studies, where we observed and interviewed players (*N* = 77) and coaches (*N* = 12) from nine organized esports clubs for two to four months per club. We explored their experiences with playful physical activities implemented during esports training. Using a reflexive thematic analytical approach, we revisited and analyzed the data.

**Results:**

The results are presented in this paper as three nonfictional short stories, creatively describing the lived experiences of the players and coaches. We emphasize patterns of shared meaning and identify the “What's” and “How's” associated with esports training when playful physical activities are incorporated. Despite differences in form, duration, and intensity compared to activities referenced in the literature on esports and performance, our results demonstrate that playful activities positively influence esports players' skills and performance.

**Discussion:**

Furthermore, our findings suggest that playful physical activities, as opposed to traditional strength and endurance exercises, often associated with physical activity, are acceptable for esports coaches. However, for maximum impact, these activities must be relevant and directly related to the games played in the esports clubs. Based on our research, we recommend implementing playful physical activities that align with the actual esports training to support developing and optimizing players' esports skills and performance.

## 1 Introduction

In recent decades, esports, also known as organized competitive video gaming (1), has transformed remarkably from a niche pastime to a burgeoning global industry predicted to expand further in the future (2). The video game-related sector has experienced exponential growth, with the number of players and audiences watching video games extending to hundreds of millions (3, 4). Players have engaged in esports either individually against the computer or collaboratively with peers, whether in school classes, college, or organized esports clubs where players collaborate to solve challenges presented in the games (1). Today, many players even pursue esports, on par with other professional sports athletes.

To develop as an esports player, continuously encountering challenges within games and experiencing the exhilarating thrill of winning battles or completing tasks within a game, training, and performance in esports is essential. During the last few years, attention has been paid to how physical activity can enhance esports players' performance, for example, by complementing esports training with physical activities (5–7). Besides enhancing performance, physical activity training affects the player's physical health and influences their mental health and sense of social belonging, especially when these activities are organized during esports training in a formal club setting, like other sports (8). Further, as amateur esports players do not reflect on physical activity and its healthy advantages in the same manner as professional players (9), it is important to include this activity in their esports training passes. In the following, we emphasize the performative part of implementing physical activity in esports training in organized clubs.

Therefore, to support and enlarge the knowledge about physical activity, performance, and esports skills, this paper is about amateur players' and coaches' experiences with physical activities implemented into esports training and how the activities influence the players' experienced performance. In this paper, we focus on playful physical activities implemented as minor physical games associated with esports practiced in clubs. However, as a Big Q qualitative research (10) emphasizing the participants' meanings and stories of and with playful physical activities, the aim is not to theoretically analyze and explain *why* and *how* the amateur players in the clubs are physically active but to use a storyteller approach (11) to show *how* the players can be physically active and *what* they physically, mentally, and socially obtain by being physically active during training and how they experience the activities' impact on their esports skills and performance. Accordingly, the association between performance in esport and physical activity is first described, followed by an introduction to playful physical activities.

### 1.1 Performance, esports skills, and personal experiences

Performance in esports is crucial at all levels, from amateur to professional (12). Whether performance is regarded as an outcome such as game scores and personal winning or as an individual task or contextual performance (mouse control, reaction time, sportsmanship, personal initiative, i.e.,) the esports players' performance, independent of the level, relies on a combination of mental, social, and physical skills (12–16) as well as the esports players' life satisfaction, mindfulness, and motivation to socialize in the game (17). Regarding physical skills, Toth et al. (15) they have shown that better motor control, such as precise finger, hand, and arm motor control among highly expert esports players, leads to better performance than players of low expertise. Based on interviews with elite esports athletes Poulus et al. (16), they found that communication and team interaction are important for a good performance. How the individual esports players in a team know each other, how they handle negative or conflicting communication, and which team bonding activities they engage in are important and impact the individual players performance.

Furthermore, personal experiences and emotions related to the esports game, such as fun, enjoyment, or rejection as a trusted player, impact the performance alongside personal challenges, social support, and general trust (18, 19). In an experiment where emotions (e.g., amusement, enthusiasm, sadness, and anger) were triggered by showing emotional video films for esports players before a match, the result showed that emotions such as enthusiasm and amusement improved their performance (18). In contrast, unpleasant emotions (anger and sadness) did not affect the players' performance.

Drawing upon a model combining competencies from digital gaming and a general model for sports competencies (20, 21) Nagorsky and Wiemeyer (14) has introduced an integrated framework encompassing mental, social, and physical skills and personal attitudes crucial for esports in-game performance. Within this framework, mental skills encompass tactical-cognitive abilities related to strategy and game prediction. These include information processing skills, including action planning, decision-making, problem-solving, memory, attention, and concentration. These mental skills find support in other studies (22–26), with Chaarani et al. (23) revealing that cognitive functions like attention and memory processing are more prominent for video players than non-video players, suggesting an association with enhanced video play and esports performance. Manci et al. (25) and Phillips and Green (26) further showed that cognitive performance differs even between games where, for example, First Person Shooters players have sustained attention, better reaction time, and inhibitions skill than Multiplayer Online Battle Arena players (17).

Social and physical skills are also critical in esports performance and are emphasized within the same framework (14). For instance, social skills such as communication, collaboration, cooperation, and physical skills related to coordination and conditioning, which encompass fine sensory-motor actions and abilities associated with endurance and speed, are vital. Various studies on esports performance further substantiate these skills (for example (15, 24, 27–29). In a study of professional esports players, Scholes et al. (27) revealed *Game Senses* (p. 8) as a crucial social skill where communication along with team cooperation was emphasized, while Trotter et al. (29) in addition to psychological skills like attention control and goal setting accentuated social support and self-regulation as factors that influence performance for esports players. Finally, other studies focusing on motor-sensory actions prove that these and other perceptual-motor abilities, like hand-eye coordination, are crucial for performance (15, 28).

Using their proposed framework of mental, social, and physical skills, Nagorsky and Wiemeyer (14) the authors have investigated skills accentuated by esports players attending various genres, such as multiplayer online battlefield arenas, first-person shooters, and real-time strategy games. Based upon a survey completed by 1,835 esports players at different levels, the authors found that they emphasized the significance of mental and social skills for their esports performance. Although physical skills were considered important, their impact on performance was relatively minor.

### 1.2 Physical activity, health, well-being, and skill development in esports

Physical skills, encompassing both coordination and conditioning, may be perceived as having a lesser impact on performance in esports due to the nature of esports athletes and their activities. Consequently, as noted Nagorsky and Wiemeyer (14), physical training does not receive specific attention in esports training. Nevertheless, research indicates that physical activity not only contributes to the overall physical health and enhancement of the physical skills of esports players (6, 30) but also plays a significant role in their mental and social well-being as well as mental skill development (31, 32). Thus, these benefits may contribute to better esports performance for the players being physically active.

At the skill level, evidence indicates a connection between physical activity and mental and social skills, although research combining physical activity with psychosocial skills remains limited (7, 33–35). Despite potential challenges in aligning esports players' reaction times in an esports context with their performance, Dykstra, Koutakis, and Hanson (35) demonstrated that the players’ reaction times improved due to their cardiorespiratory fitness during an eight-minute cycle ergometer test, suggesting enhancements in mental skills such as attention control and working memory. Other studies support the relationship between physical activity and improved mental skills, including processing speed, attention control, working memory, and cognitive flexibility (34). Moreover, physical activity accompanying esports activities has been linked to enhanced decision-making and time-planning skills in separate studies (33). Even though social skills are essential for esports players’ performance (19, 24, 29, 36), to our knowledge, there is no research concerning the association between physical activities and social skills emphasized by esports players.

### 1.3 Physical activity implemented as playful activities

Despite the established association between physical activity and its positive influence on the players' skill improvement, performance, well-being, and health, only a small percentage of esports players recognize this and integrate physical activity into their training routines for improved performance (37, 38). Surveys conducted in these studies suggest that merely 6 to 9 percent of esports players include physical activity in their training regimen to enhance performance. However, esports players’ primary motivation for being physically active appears to be related purely to physical health and not optimizing skills or esports performance.

Consequently, Danish organizations like Danish Sports Associations (DGI and DIF) have taken significant steps toward integrating different forms of physical activities into esports training at the club level for children and adolescents (5, 39), recognizing the potential that physical activity positively influences esports players' skill development potential leading to better performance. However, Gaasedal et al. (39) have shown that a mere 20% of the children and adolescents in esports clubs in Denmark are afforded the opportunity for physical activity during training, prompting questions about the reluctance of coaches to incorporate such activities. It may be a lack of knowledge of how physical activities support children's performance in esport and general health benefits from being physically active. Furthermore, the term “physical activity” is, in a Danish context, often associated with exercises like burpees, jumping jacks, or short-distance running (5). These activities scare away many esports players, as they join the esports club to practice and learn esport. Further, these activities do not bring meaning to esports coaches addicted to coaching in esport and not in physical training (5).

To enhance the esports players' readiness to engage in physical activities as an integrated part of their esports training, we chose to present and integrate playful physical activities in esports training. This form of activity promotes joy, fun, and laughter (40, 41), and according to research related to play and motivation (e.g., 42, 43) or theories of motivation, [e.g., (44)] playful physical activities motivate children to engage in activities supported by playful activities. Further, as an alternative way to introduce playful physical activities into esports coaching in clubs without scaring away players and coaches, this study includes physical activities that bring meaning for the players and coaches to enhance joy and laughter among the esports players rather than ordinary exercises emphasizing strength and endurance. To our knowledge, no research deals with playful activities and esports training or performance.

Playful physical activities such as learning games in a school setting (45), outdoor digital games (46), or the use of digital devices in combination with physical activity (47), besides triggering joy, fun, and laughter, have been shown to enhance both children's and adolescents' learning as well as their motivation for the subject taught in learning settings similar to esports training in a club. Building on collaboration, communication, and a shared agenda, which provides an adequate challenge for the players, activities for an esports context have been designed as playful physical activities (5, 48). These activities relate to the games played in esports clubs. They are considered meaningful for players and coaches, and in the same way, physical activities in learning settings are relevant for students and teachers (49, 50). Following, reflecting on the considerations according to performance, physical activity, and skill development in esport, the present study aims to elucidate if playful physical activities influence esports players' performance based on the following research questions: (1) How do young esports players' experience of playful physical activities implemented during training influence their esports performance? (2) How do the esports coaches experience the players' performance is enhanced following playful physical activities? (3) Which types of playful physical activities do the players and the coaches consider the most preferable to enhance the players' performance?

## 2 Methods

### 2.1 The context

Most Danish children under 15 are actively involved in digital gaming, from drawing books on tablets and minor games on cell phones to advanced strategy and action games on computers or consoles. Moreover, over 70% of children aged 9 to 15 dedicate over an hour to digital gaming daily (51). Despite the abundance of digital games beyond the scope of esports, the predominant genres among gamers include esports titles like First-Person Shooter games such as Counter-Strike: Global Offensive (CS:GO), Battle Royale games like Fortnite, and sports simulations such as FIFA or EA Sports FC (51).

In Denmark, children as young as eight enroll in esports clubs to include and support those who enjoy digital gaming (52). These clubs provide esports coaches to guide and enhance participants' skills and develop enjoyable challenges and tasks at an amateur/non-elite level (53). They accommodate a diverse range of children, whether they aim to engage in esports for recreational purposes and social interaction with peers or are committed to mastering specific games for competitive contexts (54). The former group, known as *casual gamers* (55), typically includes young adolescents aged 8 to 13 of both genders who enjoy various games such as CS:GO, Fortnite, Minecraft, or Roblox (56). They play at a novice, basic, or intermediate training level (57), and their participation in the esports club is primarily driven by enjoyment. They value the sense of community fostered within the esports club (58).

Another group of more dedicated esports players, termed *power gamers* (59), consists primarily of mature adolescent boys aged 13–17 years and older who are committed to a single game, which in Denmark at the time of data collection was CS:GO. They invest significantly more time in practicing and playing esports than casual gamers. They demonstrate stronger dedication to a particular game, and some aspire to pursue a career within the esports industry, akin to players from professional Danish esports teams such as Astralis, Masonic, or Estatic. Players who achieve this level and earn money for playing are referred to as *pro-gamers* (59).

Over the past five years, Danish esports clubs have emphasized health and physical activity during esports training (5). As a result, there has been widespread adoption of a shared code of ethics and health guidelines among these clubs, accompanied by numerous efforts to incorporate playful physical activities into training sessions to enhance the players' health, well-being, and development of esports-related skills. Consequently, this study focuses on children's engagement with these initiatives, and to elucidate children and their coaches' experiences with playful physical activities during esports training, we have in this study revisited data obtained from pilot studies focusing on children and adolescents playing esport in nine different esports clubs, who either had implemented playful physical activities, strives to include playful physical activities, or clubs, who didn't have included any form of physical activities in their esports training. In the last instance, the clubs were offered playful physical activities they could implement in their esports training. The research team designed the playful activities specifically for the esports players in the clubs. Examples of designed activities are unfolded in the result section.

### 2.2 Research strategy, the esports clubs and participants

In this research, a guiding principle has been utilizing a multiple-case design (60), as the study draws upon nine pilot studies or cases focusing on physical activity implemented into training within esports clubs for children and adolescents in Denmark. All clubs involved in the pilot studies had implemented physical activities during training before the study (clubs A-C, see Table 1) or intended to do so (clubs D-I, see Table 1) and were guided by the research team. The esports clubs are situated in the northern region of Denmark and were purposively chosen (61) to serve as an information-rich sample, aiming to maximize the insights gained from a limited number of cases, guided by expectations regarding their informational value (62).

**Table 1:** Overview of participants from the nine esports clubs, their ages, the coaches' experiences and employment, and the primary games played in the clubs. Lastly are examples of physical activities implemented during esports training.

Case/club	A	B	C	D	E	F	G	H	I
Number of players	8	10	9	6	12	12	5	7	8
Age of players/years	10–14	11–13	9–11	13–17	9–15	9–14	10–11	11–13	10–13
Number of coaches	2	1	1	1	1	2	1	1	1
Age of coaches (approx.), experience with gaming, employment	20-year-old male, experienced esports player, university student. 40-year-old male, experienced gamer, primary teacher	35-year-old male, experienced esports player, warehouseman.	40 + year old male, experienced esports player, IT-consultant.	17-year-old male, active esports player, high school student.	33-year-old male, active esports player, high school teacher.	40 + year old male, former esports player, mechanic. 30-year-old female; kindergarten teacher; no experience as esports player.	18-year-old male, active esports player, high school student.	23-year-old male, active esports player, university student.	37-year-old male, former esports player, case manager.
Primary game played in club	CS:GO	CS:GO	Fortnite	CS:GO	CS:GO	CS:GO	Valorant	CS:GO	CS:GO
Examples of physical activities integrated during training	Traditional children's game conducted in a gym	Ordinary exercises like push-ups and short distance running	Outdoor role play called “Fortnite Live”	Activity related to CS:GO “Knife round”	Outdoor role play called “wing man”	Outdoor traditional children's game	Activity related to Valorant “Catch the grenades”	Relay race related to CS:GO “Five-man rush”	Relay race related to CS:GO “Behind enemy lines”

Each club provided esports facilities with 6 to 14 computers and relevant software and equipment tailored for esports activities. Players utilized these resources following coaching guidance and club regulations. Additionally, all clubs were organized under DGI (Danish Union of Amateur Sports Clubs) and adhered to a code of ethics and health guidelines specific to esports, encompassing dietary habits and sleep recommendations for players, as well as encouraging physical activity (63). The clubs required players to pay for membership, and in return, they provided weekly esports training sessions lasting two to four hours. One to three voluntary coaches facilitated these sessions, offering their services free. Children attending the clubs were 9 to 17 years old, including casual and power gamers playing different esports games. Each club offered diverse physical activities during esports training, ranging from traditional children's games held in a nearby sports hall or outdoor spaces to tailored exercises focused on the primary esports game played. Table 1 details the number and age of esports players attending the clubs and information on the coaches: How many coaches are present for training in the club, short about their background, how old they are, and how many years of experience they have. Further, there is information about the primary esports games played in each club, and lastly, examples of integrated physical activities during training are listed for the nine clubs.

### 2.3 Data collection and analysis

Data from the nine pilot studies were collected between fall 2021 and spring 2023, involving multiple visits to each club spanning two to four months per club. Under the supervision of the first author, data were gathered by trained data collectors (all men aged 24 to 27 years) who had passed courses focusing on forms of qualitative data collection (covering types of observations, how to take field notes, how to use video recordings and different forms of interviews with focus at semi-structured interview). All data collectors possessed prior experience in qualitative data collection, including the conduction of semi-structured interviews with both children and adults. Additionally, during data collection, the first author guided and informed if the data collectors met any challenges regarding observations and interviews in the esports clubs.

Observation data were obtained through participant observations (64), by writing field notes, or by using GoPro video cameras, usually placed to record movements in the gaming room. If some or all players left the room intending to be physically active, the trained data collector followed the player(s) with the camera. Semi-structured interviews (65) with players and coaches were conducted in the esports clubs and were recorded on audio recording devices. Additionally, field notes were taken to complement the video recordings. Furthermore, and very importantly, the data collectors all knew of esports at different levels and had experience with gaming.

The coaches introduced the data collectors to the esports clubs, followed by a period where the data collectors mingled and played with the esport players to know the setting, the special way of interacting in the club, and to get a sense of the “spirit” of the club. This mingling aimed to create a relaxed and comfortable atmosphere in the club while data were collected. Seventy-seven young players and 12 coaches were observed during training sessions (see Table 1). Overall, 63 h and 20 min of focused observations in the form of video recordings and field notes were collected after the data collectors had mingled into the club. After the observation periods, 45 players and 10 coaches participated in semi-structured interviews. The interview lasted from 15 min for the youngest players to 55 min for the esports coaches, giving a total of 7 h and 45 min of interview data. Interview participants were as informants selected based on purposive sampling (66), as “purposive sampling is more applicable in exploratory studies and studies that contribute new knowledge” (190), fulfilling the pilot studies' aim.

Interview data from all nine pilot studies were transcribed verbatim. Subsequently, these transcriptions were revisited in the summer of 2023 and analyzed thematically using a reflexive thematic analytic approach (67, 68) to generate themes as “patterns of shared meaning underpinned by a central organizing concept” (67). The interviews were read by the first author, who also reviewed relevant field notes and observations of the implemented physical activities to supplement the information provided in the interviews.

Subsequently, the players' and coaches' experiences and their observed behaviors were analyzed inspired by Interpretative Phenomenological Analysis (IPA; 69), which seeks to “understand the innermost deliberation of the “lived experiences” of research participants” (69). Themes from the nine pilot studies were generated as the esports players and coaches shared experiences across the studies, resulting in patterns of shared meaning about the integration of physical activities into esports training—encompassing both positive and negative aspects of participants' attitudes. Several sub-themes of shared meaning across the nine pilot studies were generated based on the volume of data.

Some of the patterns of shared meaning generated as sub-themes were:

• Casual Gamer: We cannot concentrate for a long time, so a physical break is welcome—and we're refreshed and ready to do more gaming afterward.
• Power gamers: Physical activities disturb our games but are quite fun and revitalizing when we engage in them.
• Coaches: I can't see the meaning of the activity if it's not related to the esports game being played.
• Coaches and gamers: Laughter, chatting, pushing, and moving … stimulate performance.

Due to the number of interviews and the many observations of physical activities implemented during esports training, we have chosen to present the results of the reflexive thematic analysis—the patterns of shared meaning throughout the nine pilot studies—as three short stories presented in the form of creative nonfiction (70) using a creative analytical practice (CAP (71, 72);. Using CAP is in the study an attempt to “present or describe the lived experiences of those we study”, to “evoke and represent the complex emotional texture of human experiences and help [readers] to hear the heartbeats of other people, thus providing [the readers] with insights to human lived experience like none other”, and finally “enhance and enrich sense-making of the topic in question” (73). The three stories are written in the form of a *storyteller* (11), where the stories represent an analysis, a pattern of shared meaning with an emphasis on the “Whats” and “Hows”: “a storyteller chooses to craft a story to show theory. They craft a story that is highly evocative and embodied” (74).

Furthermore, we attempt to invoke a sense of liveliness and create possibilities for multiple readings and interpretations of the stories by engaging the reader differently (70). Through these three creative nonfiction forms of textualized representations, we aim to “move the reader toward a deeper understanding of a topic [esport and physical activity]”. (75). In that sense, we hope that our stories contribute to a broader conversation, discussion, or questioning on *how* physical activity could be implemented and *how* physical activity *could* take shape in terms of game-related activities, plays, and movements.

### 2.4 Ethics

All pilot projects underwent internal ethical approval following the guidelines for qualitative projects at Aalborg University. Additionally, information letters were sent to all involved esports clubs, informing coaches, players, and their parents about the project's aim, the data collector's presence, and their role as observers and interviewers. In cases of questions from coaches or parents of players, the responsible researcher (first author) answered them. Informants purposefully selected for interviews were informed of their rights as informants (76), and in the cases where the informants were children under 15, written consent was obtained from their parents. Data in the form of field notes, video recordings, and transcription of interviews from the pilot studies have subsequently been stored as confidential information due to rules at Aalborg University, which include labeling of data and password protection with AAU account details.

## 3 Results—three stories

The following three stories embrace and express many of the statements (the “Whats”) obtained from interviews with esports players and their coaches. They are supplemented by illustrative examples (the “Hows”) of physical activities implemented in the esports training sessions. The aim is to present the lived experiences of children and adolescents engaging in esports training supplemented with physical activities and, finally, to seek to answer the research question:

(1) How does children's experience of playful physical activities implemented during training influence their esports performance?
(2) How do the esports coaches experience the children's performance is enhanced following playful physical activities?
(3) Which types of playful physical activities do the children and the coaches consider the most preferable to enhance the players' performance?

Story 1 and 2 are about the esports players' experiences with physical activities and their thoughts on how it influences their performance, while Story 3 concerns the coaches' experiences and thoughts about the activities' impact on the players. During all stories, examples of different types of physical activities are displayed, and the players' and coaches' opinions are unfolded.

### 3.1 Casual gamers: it's hard to sit still and remain focused

It was time for the weekly training at the esports club. The club is located in a newly designed basement room in the middle of my hometown, which has many computers and other equipment. As my best friend Emily and I entered the esports club, the female coach, Sarah, welcomed us. “Hey, Olivia and Emily”, Sarah says while guiding Liam with something in CS:GO. … I don't like CS:GO; it's far too violent for me. I know this because Evan, my older brother, plays it at home in his bedroom. He's 14 years old. … Noah, Patrick, and Benjamin are already sitting in front of their computers, ready to play CS:GO with Liam. James, the other coach, stands behind Noah, telling him something. I think it's some hints about how to play terrorists.

“Okay, girls. What's on today's menu?” Sara smiles. She's one of our coaches—and the coach I like best. She is so friendly. “We have agreed to play FarmLife4 today  …  can you help us enter Fortnite?” Emily asks. We went to the computers and got instructions from Sarah.

After playing FarmLife4 for an hour, I go to the bathroom. I need some water and want to stretch my legs. I rub my eyes too in the bathroom and splash water on my face. It is hard to sit still and talk to a computer for so long time … Suddenly, I heard a loud noise from the clubroom, like something crashing. When I enter the clubroom, Liam is chasing Patrick. A gamer chair had been pushed over, and Benjamin and Noah were playing fighting on the floor. Benjamin yells: “You killed my man, you fool”, and tries to pull Noah to the floor.

“Kids”, Sarah shouts, separating Noah from Benjamin. They laugh and seem to find it amusing. “Now I think we all go outside. We all need fresh air”. She takes her coat and an empty tin can from a wardrobe near the door. I think we are going to play Kick the Can^1^.

It was fun … playing Kick the Can … Three rounds, and I was the last to be found almost every time. Now we are back in the club. The boys all are red in their heads after running and kicking the can. They seem to be friends again and are sitting concentrated in front of their computers, playing CS:GO together, talking in their headsets, and making plans. Olivia and I play Murder Mystery. We have a great time.

A week later, we are back in the esports club for training. It's exciting. James told us last week that he had a surprise for us today. The boys, like Olivia and me, play Fortnite today. Noah says it's awesome to try new games.

Olivia and I haven't finished our first quest in Fortnite yet when Liam bangs his hand hard on the desk. “Damn, I hate this game!” he cries and pushes himself away from the computer. I'm afraid of him when he bursts out; otherwise, he's nice enough.

“Guys, it's time to play Fortnite Live^2^”, James announces. “It's a real-time physical game, and everyone is invited”. Is it maybe the surprise he spoke about last week? I take Olivia's hand, and we go outside together.

OMG. There's a big area marked with chalk, where paper balls are placed in plastic cups and disc cones are scattered around. James tells us that we are now soldiers and war guides. Olivia, Patrick, and Noah are soldiers. Liam, Benjamin, and I are war guides. We must blindfold “our” soldiers, guide them to pick up paper balls, and hit the other soldiers by throwing them. James tells us that the paper balls are ammunition. “But”, James continues: “The soldiers must NOT step on the disc cones … they are bombs. We play to the last man standing”.

I take a scarf, blindfold Olivia, and lead her to the play zone. The game begins: “Go straight forward, Oli”, I shout, “take two steps, and you will find paper ammunition is at your right side. Throw it against Noah; he is at your left side … ”. I can see that Patrick steps on a cone disc. “Ouch, you're dead, Patrick”, Benjamin yells. Liam and I struggle to advise our soldiers to pick ammo, throw it toward each other, and, at the same time, avoid bombs. It's a matter of shouting and giving specific orders, and the two girls managed to get in touch quite well, communicating the right food, collect, shoot low at your left …

“It was a fun activity … like being in a Fortnite game”, I tell Olivia. I saw James pick up the ammo and the bombs as we went into the club together with Sarah. The boys sit in front of the computers and discuss what went wrong and what they must do next time. “Hey”, Liam says as we enter the room, “Do you girls dare to play against me and Noah? Maybe you are the best in Fortnite Live, but you can't win in a box fight”. Wow, I can't remember Liam ever inviting us to play before. “Fine with me”, I say, “Come on”, Olivia, let's knock these guys down. I can see that Sarah smiles, whispering something to James. They both smile, come to our desk, and ask if we need the codes for the LaCroix box fight.

### 3.2 Power gamer: We do not think we need physical activities, but actually, we like it

It was 5 pm, and the boys arrived at the esports club in association with the local sports association. As usual, Tom, the new young coach, was already playing on one of the computers in the esports club when the other coach, John, who worked in the daytime as a primary school teacher, entered the room. “Hey, boys”, John said while he unfolded a map of grenades related to CS:GO and invited the boys to look at the map. Soon, the boys arrived, striving for new information on how to become better gamers. They all knew that today's theme was how to use grenades. “Are you guys ready to prac some smokes from T-spawn on Mirage? Tom, do you mind showing the boys how to use smokes?”. Tom, who hadn't noticed the boys, turned round: “Hey … what did you say? smoke? Ok, follow me on the screen … ”. All the boys turned to watch the coach's in-game view projected on the whiteboard screen. The atmosphere was tense, the light was dimmed, and the boys were focused.

By 5:45 pm., most of the boys were focusing on throwing grenades. David stretched his back: “My back hurts. Hey Nolan, what about a walk?” he asked the boy beside him. Their coach, John, noticed this and shouted, “Hey boys, let's all go to the gym and exercise”. William and Jacob, deeply engaged in combat, complained, “Oh no, we are just about to challenge some FaceIt level 8 dudes in a 2v2 at Nuke. We don’t need and don’t want to exercise”, and Luke, who earlier had played in another esports club, joined in: “my former coach did not force us to get up from the chair”,. After a short discussion of the pros and cons and how pro-gamers exercised, the boys joined the others, and they all went to the gym with coach John. The new coach, Tom, stayed back in the club.

John introduced traditional exercises in the gym like jumping jacks and burpees and playing tag^3^. “The boys initially hesitated to participate, and after a while, Jacob and Luke, doing jumping jacks, exclaimed”, “It's tough and boring. Can't we go back to playing on the server?”. John shook his head and told the boys that he knew that Astralis, before a match, went to the gym to prepare themselves. “We ought to do the same”, he said. The boys continued, and by the end, the activity changed to tag; as they ran and caught up with their friends, Jacob, Luke, and the others enjoyed the physical activity.

Sweaty, laughing, and chatting, the boys returned to the club. “It was fun”, William said, “yeah”, added Jacob, “Now I’m sure ready for another esports match”. The boys returned to their computers, turned on their headsets, and concentrated on playing with and against each other and the rest of the boys for the rest of the esports training. At 7 pm the training ended. The coaches, John and Tom, checked that all equipment was left in the correct order.

The following week at the esports club, the topics were bomb planting and bomb defusal. The young coach, Tom, demonstrated these techniques on the whiteboard screen before focusing on his game. Following an hour of intense training on the Inferno map, during which the boys exchanged tactical warnings like “they're rushing B” or “they come from right”, coach John intervened, introducing a new physical activity tailored for CS:GO players^4^. Despite the usual initial complaints, the boys eagerly embraced the challenge of a relay race called Five Man Rush. Eight paper pieces representing the Mirage map, along with a “bomb” piece, were arranged on a desk at one end of the club room. Divided into two teams, the boys raced to collect the pieces along a 30-yard lane. Throughout the race, their focus was unwavering as they loudly discussed assembling the map, drawing on their experiences from actual gameplay.

As the relay race reached its conclusion, the bomb was finally planted. “Hey”, Noland shouted triumphantly, “we're finished. We won!”. With a sense of camaraderie, he exchanged high-fives with Luke and Joey. The boys nodded in agreement, their smiles reflecting the success of their pre-race strategy. Together, they returned to their screens, agreeing to a match against other boys in the club. Coach John observed their discussion on how to attack the terrorist and defuse the bomb, recollected the map they were playing, and recognized a new level of teamwork in the club.

### 3.3 The coaches' voices: social and mental benefits

#### 3.3.1 Coaches, casual gamers

Sarah waved goodbye to Patrick and Olivia, who ran towards their parents while waiting in the parking lot. “Wauw”, she said to James, “did you see that coming? Girls fighting against boys both in real life and online”. “Yeah”, answered James while he picked up a chair from the floor, “It was refreshing to see the four kids gaming and maintaining good manners without swearing and bullying as the boys often do. Maybe *Fortnite Live* was the catalyst for these new alliances. It seemed that all the children enjoyed the activity”. He collected the disc cones lying at a table in the corner.

“I’m curious”, said Sarah, wiping down tables where the children had left greasy fingerprints, “*Kick the Can* usually calm down the children, but the activity you introduced, James, seems to have a different effect on them. They worked as communities”. James went to the wardrobe to retrieve their coasts. “Well”, he said, “To win the game or survive, they had to collaborate and communicate. I guess they felt they could use those skills the next time they played Fortnite on their computer with their friends. It brings meaning for the children compared to Kick the Can”. He handed Sarah her coat, and together, they left the room. Outside the club, it was cold and dark and starting to rain.

#### 3.3.2 Coaches, power gamers

John and Tom were cleaning the club room after the training. “Hey”, began John, “why didn’t you participate in the physical exercises?”. “Well”, Tom answered as he shut down the computer he had been using, “like the boys, I am here to play CS:GO, improve my skills, and offer them advice based on my expertise. It's about practice and not wasting time on frivolous activities. Plus, we have an upcoming tournament to prepare for”. John adjusted one of the screens and set a chair upright: “I understand exercising takes time from the screen, but I noticed that the kids were more focused on playing afterward. You saw how concentrated they were in the last hour after exercising. Usually, they start to get restless towards the end of the session”. “It's hard to see the purpose of these activities”, Tom retorted, “why physical activities when they are here for esports?”. Sitting down in a gamer chair, Tom took his cell phone and opened the Twitch app. “Well”, John admitted, “today's activity seemed to challenge both brain and body. It certainly influenced Luke's behavior. After the activity, unlike usual, he played with concentration and without anger outbursts”. “Anyway”, Tom replied, “some of the guys could sure use some physical exercises. Younger but chubbier than me”. He pocketed his cell phone, stood up, and reached out for his coat: “See you next week, Super Coach John”. With that word, he left the club room.

## 4 Discussion and perspectives on implementing physical activity in esports training

In this paper, we have revisited observations and interviews with children in esports clubs about their experience with playful physical activities implemented in esport training. Following this, we have interpreted the data by focusing on the children's performance experiences based on what they were told during interviews and what was observed at their training. The results, expressed in the form of creative nonfiction stories from different contexts, differ from most literature concerning performance and physical activities included in esports training (6, 14, 77), as this literature mostly reflects quantitative research studies. Further, the physical activities included in our research are playful activities that cannot be compared with many of the activities included in the literature, such as high-intense interval training (78), ergometer cycling (35), or acute sprint exercises (79).

In the nine pilot studies, covering months of observations and hours of interviews, it is evident that the number of participants is minor compared to participants involved in quantitative research as described in the literature [e.g., (14)] and the physical activities described are not as detailed in the form of intensity, duration and time, as many of the quantitative research findings are [e.g., (78)], even that some depend on self-reported surveys [e.g., (80, 81], which can impact the validity of the study (82). However, by the decent description of the context and the players' and the coaches' experiences shown in the stories, we find it is legitimate to discuss and support our findings with findings from quantitative research.

Building upon frameworks proposed by Sharpe et al. (13) and Nagorsky and Wiemeyer (14) regarding player performance outcomes, our discussion will delve into our findings on three distinct levels of performance: an individual level with player performance in tasks or actions; a social level, considering players' social skills and abilities; and the contextual level emphasizing environmental influences on player performance. Moreover, Sharpe et al. (13) have expounded performance at the contextual level as behaviors that indirectly affect outcome performance, such as personal attitude and initiative, sportsmanship—including respect, fair play, and graciousness in winning or losing—and teamwork. This type of performance may not solely rely on skills or abilities but on individual personality differences and encompasses personal, social, and contextual performance.

### 4.1 Individual level—mental skills and external disturbance

At the individual level, task performance, including reaction time, mouse control, and response time (13), is associated with esports players' performance. These are examples of performance variables that go beyond the scope of our qualitative research. However, we found examples of behavior, such as playfighting, indicating that the players have changed focus away from esports, demonstrating lost self-control and distractions from the games, followed by decreased esports performance. Mental skills affected by this behavior include attention control, concentration, and working memory (22, 23). Without attention to these skills, the player's performance diminishes, highlighting their importance.

Further, the loss of self-control in the club context not only influences the individual player's performance but also affects the performance of other players in the club, indicating that performance depends on contextual factors such as noise and the presence of other players. A study on stressors and performance (83) supports this, as the study has found that external stressors, like audience reactions during competition, can influence the players' performance. Furthermore, using the self-determination theory Hulaj et al. (84) showed that external factors or disturbance of motivation influenced the players' overall performance, even though not significantly.

Recent reviews concerning physical activity and performance (31, 85) have examined studies involving RCT-controlled examinations of the effects of physical activities on mental skills and esports performance, considering different durations, intensities, and types of activity. For example, attention and memory, as Alho et al. (22) identified, had an impact on the player's performance and were experienced among the boys after being physically active, especially after Five Man Rush, where their knowledge of the map impacted their tactics in the coming game. Even though we observed physical activities as playful activities without focusing on duration, intensity, or type of exercise in our research, we interpreted that these activities positively influenced the players' mental skills as observed by their behavior in the club and what they said. As example, Following the activities, outer stressors in the form of children's play-fighting or running around the club were diminished, and concentration, self-regulation, and attention control seemed to be regained for all of the children, indicating the same relationship as found by authors researching mental skills, physical activity, and esports performance [for example (34)].

Physical activity breaks in six minutes of light-intensity walking in esports competitions have positively impacted the player's executive functions and personally experienced performance (33). Furthermore, in contexts other than esports, breaks of different intensities, durations, and types have been shown to influence mental skills, such as the ones used in esports training. In schools, for instance, where cognitive abilities are used and “trained” daily, it has been s demonstrated (86) that physical activities in the form of activity beaks, brain breaks, or energizers—defined as short bouts of physical activity performed as a break from academic instruction—positively influence the children's cognitive abilities. Similarly, other forms of physical activity, such as curriculum-focused classroom-based activities or physically active lessons, have been demonstrated to impact school children's performance (87, 88). This indicates that breaks from ongoing cognitive tasks, such as classroom education or esports training, are equally or even more important than the duration and intensity of the break and type of activity or exercise.

**Figure 1:**
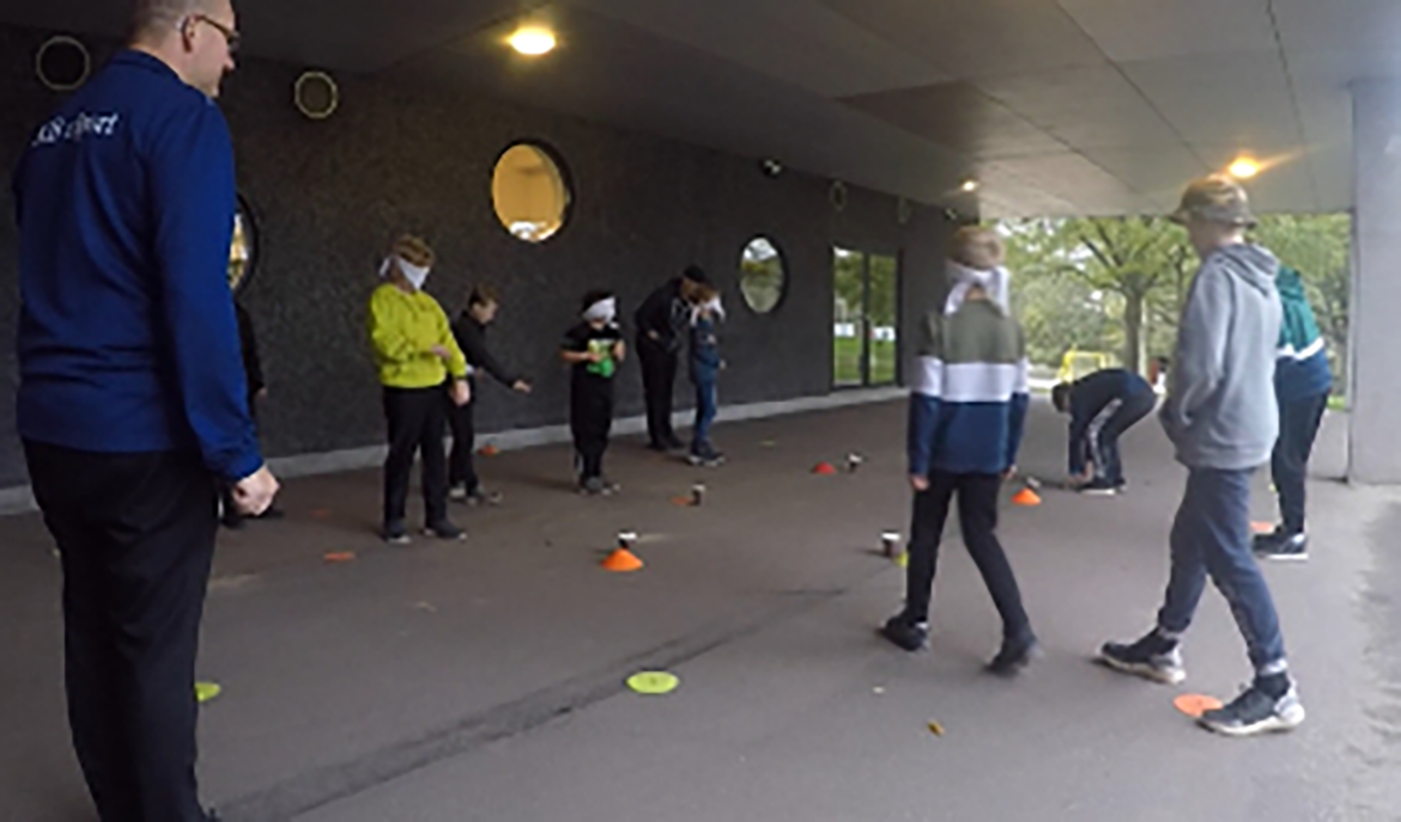
Kids are playing Fortnite Live near an esports club. One is a War guide, standing behind the line, while the other acts as a Soldier—blindfolded and supposed to follow commands from their War guide.

However, the relevance of physical activity related to the specific tasks children are engaged in appears to have an impact; the “meaningfulness of the activity” plays a significant role. In school settings, where students, like in esports training, may experience cognitive overload during the day, meaningful physical activities are beneficial compared to non-curriculum-relevant activity breaks like brain breaks (89). Similarly, we found that meaningful physical activities during esports training, such as “Fortnite Live” (see Figure 1) or activities designed for casual gamers, positively affected the players' mood and engagement more than activities unrelated to esports games, such as Kick the Can or Jumping Jacks. Furthermore, coaches’ attitudes were influenced by the meaningfulness of the activity as they recognized that relevant breaks away from the computer emphasized the development of skills related to games, such as communication and collaboration. Moreover, motivational factors such as children's knowledge, autonomy, competence, and relatedness to a task (such as knowledge of a map and ability to collaborate (44, 90); during esports training can be stimulated by meaningful activities, which enhance skills necessary for gameplay and increase engagement in subsequent games.

### 4.2 Social level—communication, collaboration, and teamwork

Social skills such as communication, collaboration, and teamwork, identified as elements in the playful physical activities as elaborated in their stories, are related to the social level of performance, where the players' social competencies impact their performance (13, 14). In addition to these personal skills, the social support the players receive, whether functional or structural, influences their performance (29). Social support in the form of emotional connection, esteem, and tangible support (91, 92) affects individual player performance, highlighting the importance of the social dimension in physical activities.

**Figure 2:**
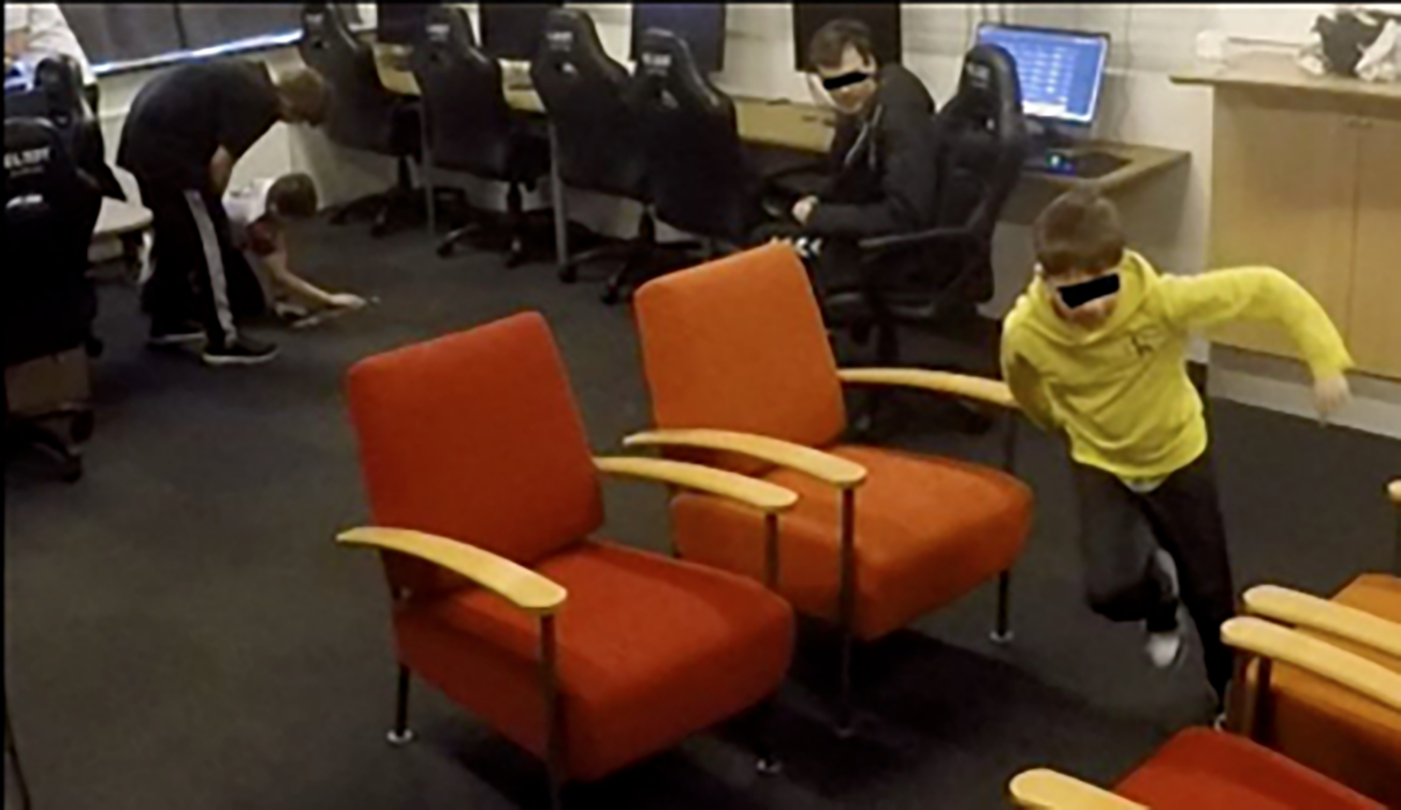
The boys are playing Five Man Rush in an esports club. The boy in a yellow shirt is running for yet another piece of the map while his teammates in the back are trying to puzzle some already-found pieces together to form the Mirage map.

Physical activities like core training, cardiovascular training, or strength training do have an effect at the individual level (6), but to impact the social level, it is necessary to unfold the physical activities as social physical activities. Activities like Kick the Can and Relay Race are social activities and, as we have demonstrated, are valued by the esports players as breaks in the training routine. Combining these activities with relevant tasks, as in Five Man Rush (see Figure 2), where the activity included fast running as well as a mental exercise in the form of map knowledge and a memory game including collaboration and communication, can make the physical activity even more challenging at different levels and contribute to the stimulation of a variety of mental and social skills important for esports performance at the same time.

### 4.3 Contextual level—enjoyment, happiness, and fair play

Laughter, respect for opponents, fair play, and graciousness in winning or losing were observed during the players' playful physical activities. According to Sharpe et al. (13), these factors relate to the contextual level of performance. As indicated in the stories, due to the physically playful activities, the atmosphere of the club and among the players changed from a tense to a more relaxed atmosphere. Normative beliefs and stressors like friends' and teammates' beliefs and management problems within teamwork have been demonstrated to influence the players and their performance at the contextual level (93, 94). Playfulness, enjoyment, and happiness, as factors associated with playful activities, are recognized in everyday life and school (95–97) and work environments (98). These factors manage stress and other disruptions, supporting playful activities in esports training to reduce “noise”, such as negative comments from teammates, playful banter from other players, and various stressors encountered in the club setting.

Although many studies advocate that physical activity positively influences the players' performance (6, 7, 31), and we in this paper have demonstrated that physical activities implemented playfully likewise impact the players' performance—contributing both at the individual, social, and contextual levels—there is still seen resistance among physical activities implemented during training among both esport coaches' and players as shown in the stories. In Denmark, although esports clubs have implemented a code of ethics and health policies to encourage esports players to be physically active (63), only one in five are offered physical activities during training (39). Therefore, to encourage esports coaches to implement physical activities in esports training to stimulate the skills and enhance the performance of esports players, it is necessary to inform them about the potential of implementing playful activities during esports training and further assist and help the coaches by for example teaching them how to implement activities during training. Moreover, new relevant, playful physical activities targeting players and games played in the esports club to ensure the relevance of the activity can be developed and designed in collaboration with the esports coaches.

### 4.4 Challenges and future research

Denmark's organized sports clubs, including esports clubs, have been established and formed to promote social interaction (54). Within this context, playful physical activities can be seen as a tool to promote socialization, health, and physical activity among esports players. However, implementing and exploring these playful physical activities come with challenges as they are different approaches to conceptualizing “physical activity”. As expressed in the stories, challenges arise for both the players and the coaches. Questions related to “What is the meaning and what is the purpose?” are expressed by both parties. Here, it is important to think carefully about implementing and articulating playful physical activities, as it could create a dislike for esports, given that players might have chosen esports due to its “lack” of physical activity. Some esports players may associate physical activity with more traditional sports and find esports clubs a safe space within the community because of this particular “lack”. This stresses the importance of *not* connecting the implemented activities with common sports and health-related activities but emphasizes the joy and playfulness of the activities and the relatedness of the games played.

As initiatives involving physical activity in Danish esports clubs are a relatively recent phenomenon, we recommend future studies explore how clubs integrate, for example, playful physical activities and follow such communities for a longer period with a holistic methodological setup to understand more perspectives within these clubs, e.g., players, coaches, board members, parents. We would also welcome future research exploring how esports federations developed and conceptualized health and physical activity in the esport-specific context.

Further, as this study has explored young amateur players (casual and power esports players) and their coaches' experiences with playful physical activity, it would be too ambitious to regard these findings as comparable to, for example, older pro/elite players/athletes. Nevertheless, it raises important questions for elite organizations and communities regarding the possible performance benefits from alternative physical activities to contribute to team cohesion, communication, and a sense of belonging.

## 5 Conclusions and recommendations

In the present study, based on data from nine pilot studies, we investigated how playful physical activities, as an alternative to traditional physical exercises such as strength or endurance training, implemented during training in organized esports clubs, influence players’ experience of their performance. Addressing research questions about how young players and their coaches experience playful physical activities implemented during training, we found that the activities positively influenced the players' individual, social, and contextual performance. They felt more energetic and experienced that their collaboration was improved. Further, they experienced that the environment in the club was more relaxed and friendly, following playful physical activities.

Supported by primary quantitative research results regarding esports, performance, physical activity, and esports skills, as well as other findings related to physical activities in school settings, we further demonstrate that these forms of playful physical activities not only enhance esports players' skills at the mental, social, and contextual levels, leading to improved performance but also induce engagement and motivation for playing esports. Furthermore, these playful physical activities bring laughter, joy, and fun to the esports club, influencing the atmosphere. Based on the findings, we recommend that coaches in organized esports clubs and other settings design, plan, and implement playful physical activities during training to improve the players' engagement and motivation for the games. Further, as playful physical activities support esports players in optimizing esports-related skills, this can lead to improved performance and probably continuous interest in developing esports.

## Data Availability

The raw data supporting the conclusions of this article will be made available by the authors, without undue reservation.

## Footnotes

^1^Random activity observed during esports training in one of the clubs with primary casual gamers.

^2^Activity for Fortnite. Designed by an esports coach in one of the clubs.

^3^Random activities observed during esports training in one of the clubs with primary power gamers.

^4^Activity for CS:GO. Designed by researchers.
